# LSAT: Liliaceae Simple Sequences Analysis Tool, a web server

**DOI:** 10.6026/97320630014181

**Published:** 2018-04-30

**Authors:** Manosh Kumar Biswas, Sathishkumar Natarajan, Dhiman Biswas, Ujjal Kumar Nath, Jong-In Park, Ill-Sup Nou

**Affiliations:** 1 Department of Horticulture, Sunchon National University, Suncheon, Jeonnam, 57922 South Korea; 2 Department of Computer Science and Engineering, Maulana Abul Kalam Azad University of Technology, WB, India

**Keywords:** SSR-marker, Web-Tool, Lily

## Abstract

LSAT is a web-based microsatellite SSR marker designer tool specific for the Liliaceae family. It is developed using HTML, CSS, PHP, Perl and Java scripts. It works without extra add-ons on standard browsers. LSAT provides SSR primer designing service using the web interface. It helps in SSR mining and primer design. LSAT is user friendly with customizable search parameters producing visual output having download options. The current version of LSAT is backed by two data sets, namely, lily EST (Expressed Sequence Tag) from NCBI and lily nr (non redundant) with 4,099 and 216,768 unigenes, respectively. LSAT will be updated regularly upon availability of additional data (either EST and/or transcriptome) on Liliaceae.

**Availability:**

LSAT is available for free at http://210.110.86.160/Lsat/Lsat.html

## Background

Microsatellite markers are extensively used in plant breeding due to its co-dominant nature, genomic abundance, and high degree of polymorphism. With the advancement of sequencing technology, large number of sequences from wide range of plant species is made available in the public domains. This is a valuable resource for designing SSR marker. It could be a potential tool for QTL mapping and marker-assisted breeding of Lilium sp. High polymorphic SSRs were developed and validated for genetic diversity and population structure in lily accessions study [1]. Development of hyper variable, effective, polymorphic marker is a big challenge for the molecular biologist. Several SSR analysis tools are available for developing such type of markers. However, there are still many issues specific to SSR mining and primer design. Most of the tools are command line specific with lack of graphic interface with little statistical information on SSR. Among them, some of the tools are stand-alone, that could run locally or with the help of web-based tools like MISA-web (MicroSAtellite identification tool) [2]. Most of them are platform dependent (viz. SSR locator, IMEx) [3] and compatible with either to windows or to LINUX/UNIX system. These include Repeat masker [4], Sputunik and REPuter [5], SciRoKo [6], TROLL [7] and MISA. The major drawback of web-based SSR tools is data limit for uploading large data file with maximum 2 megabase (Mb) (http://webblast.ipk-gatersleben.de/misa/) and restriction to use as online SSR picking tools. Therefore, we describe a unique web-based LSAT tool, which is easy to handle, user friendly for SSR development. It also provides graphical user interface option for customizing search parameter, and output results with detail statistics can be downloaded.

## Methodology

### Software Input

**Figure 1:**
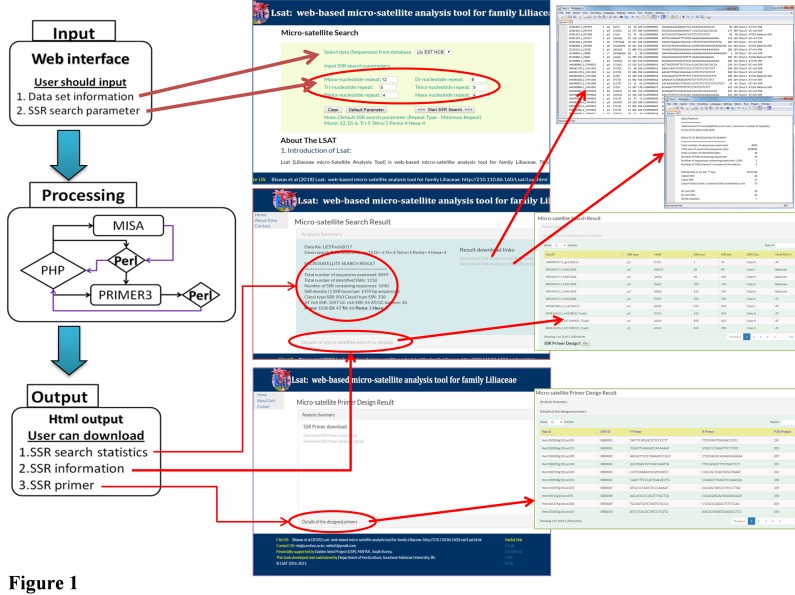
An overview of LSAT database operational mode for SSR primers selection from the nr (non redundant) databases in the public domains. Description on the input and the output of LSAT with the data processing scripts and features for retrieving SSR for Liliaceae family is shown.

LSAT input interface was developed using HTML and JAVA script. LSAT is backed by two main data sets as shown in Figure 1. The interface has options for selecting a desired dataset and custom select parameters (motif length etc.) in addition to default setting. It should be noted that the SSR search should be run for submitting input information to the server for processing when custom parameters are used.

### Software Output

The customized input information is received by a PHP script to generate the SSR search parameters file and the sequences data file together to subsequently invoke MISA for running SSR search. The modified version of the MISA script generates two output files. One with statistical summary and another with SSR information like SSR characteristics and 200 bp flanking regions in either side of each SSR locus. The PHP script will generate a HTML page with visualized SSR results for download. The interface further prompts users for primer design. Thus, a PHP script runs primer3 (http://primer3.sourceforge.net/) with the help of several PERL scripts loaded on to the system to design SSR primer. Thus, the designed SSR primers are visualized as an output.

### Caveat and future development

LSAT is freely available for use. It is a user friendly, fast and a robust web-server of SSR marker specific for Liliaceae family. It provides a Graphical User Interface (GUI) with customized parameters of SSR design with known statistical information. This is the unique feature of LSAT compared to other existing SSR tools. The information on SSR will help fast screening of SSR primers prior to help in wet-lab validation. We plan to improve LSAT with increased the number of datasets on SSR at the backend. We also plan to add user requested datasets to the interface. The interface will be upgraded frequently with advanced scripts by displaying results in graphical representation having advanced features for SSR marker.
